# Middle Eastern Conflicts: Implications for Refugee Health in the European Union and Middle Eastern Host Countries

**DOI:** 10.1200/JGO.2016.005173

**Published:** 2016-06-15

**Authors:** Michael Silbermann, Michel Daher, Rejin Kebudi, Omar Nimri, Mazin Al-Jadiry, Lea Baider

**Affiliations:** **Michael Silbermann**, Technion-Israel Institute of Technology, Middle East Cancer Consortium, Haifa; **Lea Baider**, Assuta Medical Center, Tel Aviv, Israel; **Michel Daher**, University of Balamand, Saint George Hospital, Beirut, Lebanon; **Rejin Kebudi**, Oncology Institute, Istanbul, Turkey; **Omar Nimri**, Ministry of Health, Amman, Jordan; and **Mazin Al-Jadiry**, Children's Welfare Teaching Hospital, Medical City, Baghdad, Iraq.

## Abstract

Until very recently, health care in conflict settings was based on a model developed in the second half of the twentieth century. Things have changed, and present civil wars, such as those that are currently taking place in the Middle East, do not address the complexity of the ongoing armed conflicts in countries such as Syria, Iraq, and Afghanistan. These conflicts have caused a significant increase in the number of refugees in the region, as well as in Europe. Hundreds of thousands of refugees succeed in settling in mid- and north-European countries, and their health issues are becoming of great importance. Refugees in Europe in the twenty-first century do not suffer so much from infectious diseases but more from noninfectious chronic diseases such as diabetes, cardiac disease, and cancer. These facts profoundly alter the demographics and disease burden of hostility-derived migrants. Thus, host European countries face situations they have never faced before. Hence, new approaches and strategies are urgently needed to cope with this new situation. The efforts to absorb refugees of different traditions and cultural backgrounds often cause increasing ethnic and religious tensions, which frequently escort the emergence of social violence. To date, little attention has been paid to the overall load of distress being experienced, especially among the first-generation refugees. The current ongoing hostilities in the Middle East induce a long-term health impact on people expelled from their homes, communities, traditions, and cultural environment. The realization of collective suffering forces communities and governmental health agencies to develop new programs that include social determinants to overcome the severe cultural gaps of the newcomers in their new European host countries.

## INTRODUCTION

In recent years, the migration movement from the Middle East and North Africa (mainly from Afghanistan, Iraq, Syria, Turkey, and the Maghreb) has increased, bringing the refugee population to nearly 10% of the total population in several countries in the European Union (mainly in Western Europe and Scandinavia). One of the formidable challenges facing the receiving countries is the management of the refugees’ health needs, because they comprise a vulnerable population healthwise and may present the perception of a different disease profile from that of the receiving population.^1^ Western societies recognize their moral obligation to extend effective health care services to all their inhabitants, including migrants; yet, language and cultural barriers, especially within the first-generation migrants, tends to lead to lower attendance for screening tests, such as mammography and tests for cervical cancer.^1^ Although documentation shows that newly arrived immigrants are healthier than the recipient population, in time, the healthy migrant effect may wear off.^2,3^

## CURRENT REFUGEE HEALTH STATUS IN WESTERN EUROPEAN COUNTRIES

A recent Danish study found that among a group of newly arrived refugees, 65% had one or more severe somatic health problems, ranging from liver carcinoma to tuberculosis and diabetes. Refugees are, therefore, in special need of screening programs.^4^ Other European studies have shown increased mortality rates among migrants compared with natives for cancer of the liver, esophagus, and stomach, although low mortality rates for breast and lung cancer.^5^

In Norway, refugees frequently use emergency primary health care services for undiagnosed pain. Conceivably, mental distress may be the cause of this complaint as a result of cultural and linguistic communication barriers.^6^ Indeed, the mental and psychosomatic well-being of immigrant populations promotes their successful integration into the new society. Factors negatively influencing the mental health of immigrants are economic instability, cultural and social marginalization, racial discrimination, and lack of statutory documentation.^7^

Studies from Sweden indicate that although several resources for changing the migrants’ lifestyle are available, these programs are not culturally adapted.^8^ For example, a challenge that arises when trying to implement an adaptation program would be that of motivating women to participate in mammography screening programs. Any interventional program would have to take into account not only evidence-based elements but also Middle Eastern cultural and lifestyle habits.^8^

A literature review of Afghan refugees and asylum seekers who resettled in industrialized nations pointed to a high prevalence of depressive and post-traumatic symptoms as a result of eroding cultural values that dictate family affairs.^9^ The process of being uprooted may lead to culture shock,^10^ namely, feelings of alienation, isolation, and depression.^11^ Similar findings were reported from the Netherlands.^12^ Such phenomena are found mostly in more recently arrived refugees, probably as a result of poor health literacy and understanding of the local health system, along with medical distrust and discrimination.^13^ US studies found that the duration of residence in the receiving country resulted in increased exposure to discrimination.^14^ This association was significant in first-generation immigrants but not among their descendants.^15^ A review of the healthy migrant effect in Denmark showed that refugees and family-reunited immigrants had lower hazard ratios of breast cancer and stroke 5 years after arrival compared with Danish-born citizens. However, the ratio reversed to higher hazard ratios thereafter.^16^

A recent report from Italy indicated that, overall, cancer mortality among immigrants was lower than among Italian citizens. However, mortality was higher for liver cancer in immigrants from Asian and sub-Saharan countries (possibly as a result of exposure to the hepatitis B virus and to aflatoxins), for lung cancer in immigrants from Asian countries (because of smoking habits), and for cervical cancer in immigrants from East European countries.^17^ A recent report presenting the findings of a study comparing cancer diagnoses among refugees (n = 20,642), migrants (n = 3,395), and domestic inhabitants in Slovakia (n = 11,643) showed that the incidence of cancer in refugees (2.6%) and migrants (1.89%) was higher than in the general population (0.97%). Among the highest risk group—the refugees—the standardized incidence ratio for cancer was 0.57% among women and 0.44% among men. In the combined migrant/refugee population, the incidence of cancer increased to 5.1% in patients coinfected with HIV, viral hepatitis, and human papillomavirus. Cervical, vulvar, lung, stomach, and breast cancers and lymphoma were among the most common malignancies in this population. Moreover, in a 3-year cumulative study, mortality rates among the refugees and migrants were 0.839% and 0.681%, respectively, compared with 0.437% in the general population study group.^18^ Most migrants are young people who usually suffer much less from cancer. In the United States, Israel, and other Western countries, childhood cancers comprise only 2% of all cancers.

## REFUGEE RESIDENCE STATUS IN THE MIDDLE EAST

One of the major entry gates of Middle Eastern refugees into Europe is the Turkish-Greek border. The refugees go through detention centers on the Greek side of the border. In 2010, the European Agency for the Management of Operational Cooperation at the External Borders of the Member States of the European Union (FRONTEX) reported that Greece accounts for 90% of all detections of illegal border crossings in the European Union.^19^ The main problem in all visited detention centers was the substandard hygiene conditions, especially because of overcrowding and the lack of personal hygiene facilities.^20^ These conditions have not changed since then. Considering the traumatic experiences that many migrants undergo, clearly, more psychosocial support should be provided and sustained.

Today, more displacement takes place in urban and middle-income settings. Consequently, an epidemiologic shift away from communicable diseases that have historically characterized refugee populations has occurred. In light of the increase in noncommunicable diseases (NCD) faced by refugees, a recent survey was undertaken to explore the prevalence of NCDs among Syrian refugees in noncamp settings in Jordan. More than half the refugee households reported one member with an NCD. A significant minority did not receive care, citing cost as a primary barrier.^21^

A study gathering basic demographic and clinical data about cancer in refugees in Jordan (mainly of Iraqi and Syrian origin) between 2010 and 2012 and refugees in Syria (all of Iraqi origin) between 2009 and 2011 revealed that cancer is an urgent public health problem in refugee settings, thereby augmenting the scarce reported data about refugees with cancer.^22^ Data from national, representative, cross-sectional surveys of Iraqi refugees in Jordan and Syria in late 2008 and early 2009 showed that most respondents in both countries perceived health care as unaffordable, and cost was an important barrier to care.^23^ A recent census conducted in Jordan (O. Nimri, personal communication, February 2015) showed that there are 1.3 million Syrian refugees in Jordan, and 34% of them are living in Amman. The Jordanian National Cancer Registry reported increased numbers of Syrian patients among refugees who were registered and treated for various types of cancer.

In less than 2 years, Syrian refugees constituted approximately 15% of the Jordanian population, resulting in failed social, health, education, housing, energy, and water provisions, unemployment or low wages, and many other deficiencies. This affected not only the refugees but the local population as well, sometimes with provoking consequences.

Lebanon has hosted approximately 1.1 million Syrian refugees. There, too, Syrian refugees arrived with chronic and costly illnesses, such as diabetes, heart disease, and cancer. The international humanitarian agencies that provide aid for health needs for refugees simply do not have enough funds to treat these diseases. According to a report from the Office of the United Nations High Commissioner for Refugees regarding cancer care for refugees in Jordan and Syria between the years 2009 and 2012, data for approvals and funding for 511 applications for cancer treatments were evaluated, and only 48% were approved and funded. Moreover, for those approved, the full amount was given only in about 70% of patients. The main reason for denial (52%) was poor prognosis. Presumably, lack of funds was one of the main reasons for the denials.^24^ More than 2 million refugees, mainly Syrians, have gone to Turkey. In Turkey, all patients registered as refugees according to the Law on Foreigners and International Protection, which regulates all proceedings for refugees living in Turkey, have been provided with free medical treatment as Turkish citizens.^25^

The overall health care situation, and particularly cancer care, is expensive and drains huge amounts from the national health budget. This problem had been dealt with to some extent with the help of nongovernmental organizations and other international charity organizations that contributed by paying the treatment invoices. This source, however, came to an end during the current difficult global situation, exacerbating the Syrian refugees' situation further. The refugees fleeing to Europe today face a totally new way of living within communities with different backgrounds, racial ethnicities, cultures, traditions, religions, languages, and economic barriers.

The situation of pediatric patients is not different from that of the geriatric population. An updated survey recently carried out in Turkey indicated that most refugee children affected by cancer were diagnosed when the tumor was already at an advanced stage, whereas some came with relapsed disease.^26^ Most of the children came from Syria, and the rest came from Iraq, Iran, and Afghanistan. The estimated number of new childhood cancers among the refugees in Turkey ranged between 60 and 100 annually. Overall, the incidence rate of pediatric cancers among the refugees was similar to that of Turkish children. The patients, their families, and the medical teams were faced with the following problems:

- Shelter
- Social and psychologic problems in adapting to a new setting
- Language barriers
- Compliance with therapy
- Hygiene (personal hygiene; hygiene at home, namely hand washing; clean food; clean water; adhering to hygiene rules)
- Communication problems within families and with health care personnel
- Lack of donors for transfusion, especially in transplantation patients, where the number of transfusions needed is high
- Difficulties in accessing medication as outpatients (therefore, patients must be hospitalized longer)
- Poor prognosis because of advanced disease at diagnosis and relapsed disease (delay in diagnosis, unable to come in time because of conflict/war in the area)
- High incidence of hepatitis C seropositivity in patients who were multitransfused previously (inadequate screening for infections in blood banks in their home countries)
- Financial problems

Nevertheless, the outcome of the treatment that patients received in Turkish hospitals was encouraging, with survival rates close to those of Turkish children.

## WHAT IS THE OVERALL SITUATION REGARDING NCDS AMONG REFUGEES?

The United Nations High Commissioner for Refugees recently reported that there are approximately 40 million displaced people, including refugees, internally displaced people, and migrants, seeking asylum worldwide.^27^ In this context, NCDs have received little attention despite their overall burden.^28,29^ Unlike refugees fleeing conflicts in Africa, where diseases of poverty, such as diarrhea, malaria, or cholera, take their toll, Syrian refugees are more afflicted with chronic and costly NCD diseases, such as diabetes, heart disease, and cancer. According to a Disaster and Emergency Management Authority survey, approximately 10% of refugees report problems with NCD diseases, including hypertension, diabetes, and cancer.^30^ Cancer and cancer care are increasingly recognized as a major worldwide challenge, given their global, financial, social, and health implications.^31^ Treatment of cancer in refugees is also of increasing importance, because it places a substantial burden on the health systems of the host countries. An analysis of cancer care in low-income refugee settings is needed to inform future responses. Such diseases present specific challenges for refugees, including continuity of care, the need for expensive treatments, the need for skilled physicians, regular screening and follow-up, and avoidance of treatment interruptions as a result of displacement.^32^

## ETHICAL RESPONSIBILITY OF HOSTING COUNTRIES

Legal and illegal immigrants form a large and disputed group in many countries. These migrants include refugees, asylum seekers, immigrants, undocumented workers, or, as in French, *sans papiers.* These people include men, women, children, agricultural workers, domestic workers, computer programmers, health care professionals, and many others. Their existence raises an important ethical question: do societies have an ethical responsibility to provide health care for undocumented and documented migrants on an equal basis with their citizens?

Ethical traditions of medicine and health care focus on a patient’s needs and should not discriminate among patients. The Hippocratic Oath taken by physicians, nurses, and health care professionals includes the following: *“*The health of my patient will be my first consideration; may I never see in the patient anything but a fellow human in need. I will treat all patients with compassion, no matter how much they differ from me. I will respect the secrets patients confide in me.*”*

There is nothing in the oath that excludes illegal immigrants or poor citizens from care because of any constraints. Our commitment to our ethical principles requires that any patient who is sick deserves treatment, regardless of personal profile or legal status.

Moreover, if we used our health care system to police illegal immigrants, it would undermine the trust between patient and physician and, by extension, trust in the health system at large, and would make such people even more reluctant to seek health care services. This could worsen the patient's own health and, at times when patients suffer from communicable diseases, could be harmful to others, as well. Furthermore, issues related to illegal immigrants should be resolved through appropriate policies and laws, not through the health care system.^33^

Concerning the issue of migrants and our ethical responsibility, we face the following questions:

- Does migration lead to unjustified disparities in health care? In other words, does denial of health care to undocumented migrants amount to wrongful discrimination?
- The equal treatment of patients is one of the core values in professional medical ethics. The Geneva Convention calls for treatment without any “adverse distinction founded on sex, race, nationality, religion, political opinions, or any other similar criteria.” Although questions about migration often refer to issues related to ethnicity and race, migration involves specific barriers (legal status and language issues) and, consequently, requires unique educational requirements, referring to human rights.
- Do countries that provide universal health coverage for their citizens provide the same health care for all migrants?

There are two opposing views regarding the rights of migrants. Some argue that illegal immigrants have no claim to health benefits because people who have no right to be in a country have no right to the benefits in that country. Others hold that access to care is a basic human right and should be provided to everyone—recognized citizens and illegal immigrants alike. Neither view, in our mind, is adequate. What is needed is a balanced approach that considers the problem of illegal immigration on one hand and adequately values the complexity of mortality on the other.

Lastly, societies have often used marginalized and vulnerable people—minorities and migrants—to do the most disagreeable and difficult labor. Although some laws prohibit the employment of undocumented migrants, the economic system encourages and relies on them.^34^ It is time for societies to come up with a balanced approach to regulate this social responsibility.

Do rich countries have a moral duty to provide health care for sick people outside their territory? Global justice emphasizes the need to assist because it serves human purposes well. The current political milieu promoting global justice is complex, because it involves both social justice and meaningful autonomy leading to more peaceful relations regionally and globally. To reach this goal, we must retain good political judgment alongside a willingness to experiment.^35^

We have touched on two urgent issues: illegal immigration and health care. We fully realize that readers may have different views of the meaning of social justice. Our aim is merely to provide an argument for the critical role of social justice. We believe that global justice embodies the principle of basic equality for human beings while reality reveals the inequalities in health and should be rectified.^36^ Refugees living in marginal urban or suburban communities become alienated from the mainstream Western society and create enclosed safe communities that survive separately from the liberal mainstream.^37^

Can the historically homogeneous and stable European society incorporate such cultural diversity? Or will immigration deepen cultural tensions, social instability, intracommunal conflict, and group alienation? This challenge is particularly relevant when examined under the prism of actual conditions of limited health services, educational disparity, and wide economic gaps. There is a sense of despair because there is no prospect of establishing any semblance of the former culture for oneself, one’s family, or community,^38^ whereas religious belief functions as a source of exclusion, segregation, and alienation.^39^

Beyond the significant humanitarian aspect of this crisis, forced displacement and population transfer on the basis of ethnic and sectarian identities is reconfiguring the political maps of Europe and threatening to unravel the prevailing order of stability. Refugees are pushed to the fringes of society and are unable to reconstitute their customary family existence or to earn a gainful living under minimal human standards within their precarious daily lives.^40^

In sum, any health interventions must be tailored on the basis of the immigrants’ ethnic-related risk factors and reception factors of the society to which they migrate. Increasing awareness about cancer disparities is the key to providing access to quality medical care.^41^

## DISCUSSION

Currently, more people are displaced by conflict than at any time since World War II. The ongoing war in Syria has led to the worst humanitarian crisis of the twenty-first century so far. More than 7 million people have fled the Syrian conflict, entering neighboring countries—mainly Turkey, Lebanon, Jordan, and Iraq—and, thereafter, hundreds of thousands have sought asylum in countries in the European Union.

Europe is experiencing one of the most significant influxes of migrants and refugees in its history. Caught in civil war, terror, and poverty, and drawn by the chance for a better life, hundreds of thousands of refugees have fled the Middle East and Africa, risking their lives on a voyage to the unknown.^42^ Europe, among all world regions, has hosted the largest number of international Muslim migrants representing nearly a tenth of Europe's total population.^43^

The number of refugees entering Europe passed the 1 million mark in 2015, a long-expected and symbolically significant capstone to a year in which displaced persons flocked to the continent in historic proportions. In a recent study of the availability of antineoplastic medicines, the European Society for Medical Oncology found a marked decrease in access to drugs, mainly the more expensive, newer drugs, but also the inexpensive, older but essential medication. Because of their displacement, race, and language barriers, refugees and migrants do not have access to drugs, treatment, or health clinics. With limited access to the health care system in their adopted countries, refugees and migrants neglect early signs and present only when severe symptoms oblige them to search for some form of medical help.^44^ The international humanitarian agencies that provide for refugees worldwide do not have the funds to treat chronic diseases, leaving many without access to medical or palliative care. Almost half of the patients present at a late or terminal stage of illness. Half do not begin or complete therapy, and most do not return to the hospital. Death is the expected outcome.

Do nationality, personal identity, race, and social status affect early diagnosis? Are there any current multilingual health programs for deterrence, cancer prevention, accessibility to treatment, or any qualitative palliative care for pain control and care for terminal illness? The answer is that there are wide discrepancies in cancer care among European countries, which are even more pronounced for refugees and immigrants.

Access to minimal quality cancer care is now a fundamental medical issue in all of Europe. There is great heterogeneity in the availability of basic medications, prevention programs, and economical health resources among the host countries. Surprisingly, however, despite the profound effect of war and forced migration on people's living conditions, relatively little attention has been paid to both the physiologic and psychological impacts of being a refugee.

What is driving people from their home countries? They move to escape war, persecution, unemployment, poverty, and environmental degradation. What is pulling them to the destination countries? They move to find better opportunities, provide more support for their families, and build safer lives. Yet, with the unprecedented migration to Europe seen in recent years, policies limiting access to health care have become prevalent. Barriers to health care for illegal refugees go beyond policy and range from financial limitations to discrimination and fear of deportation.^45^ In conversations with migrants (M. Al-Jadiry, personal communication, December 2015), it becomes apparent that most refugees do not leave their countries to obtain better health care, but to flee ongoing military hostilities, violence, or persecution. Reportedly, the incidence of cancer changes to the level of the new host country in one or two generations. In addition, it has become evident that children and young adults who entered Europe in their 20s remain at low risk, similar to that in their original country, whereas children born in a high-risk country (such as most Western European countries) are destined to be at high risk for the rest of their lives. These data are highly relevant for cancer prevention policies.^46^ Yet, there is still a paucity of research regarding the profile of cancers among displaced populations, specifically Middle Eastern and African refugees in Europe. Similarly, Middle Eastern countries have welcomed millions of refugees in the past decade, first Afghans, followed by Iraqis, and now Syrians. The massive entry of refugees to countries such as Turkey, Jordan, Lebanon, and Iran posed a substantial burden for the hosts’ national health care systems. Conversely, the inability to obtain medical attention has led to significant delays in seeking the correct treatment.

In facing the immigration problem, the European Union generally and Germany in particular serve as models promoting intercultural education as a means of integrating immigrants into their new society. The intention of integration was to leave the immigrant's personal ethnic and religious cultural identity intact while integrating into a public European community.^47^ More recently, the concept of integration embodies the notion of European interculturalism, because it was realized that something intercultural was already happening due to external and internal forces, and yet, it needed to be managed by means of an intercultural dialogue. In the European intercultural discourse, the immigrant is exposed to European culture and learns to embrace it. In so doing, the newcomers also become more familiar with the local health care system and the availability of services that they are entitled to receive. The hope is that in this way, the cultural gap between Middle Eastern migrants and the local European population can be bridged. Yet, the challenges of integration go beyond the enormous tasks of teaching the language and finding jobs. Germans who are in direct contact with new arrivals talk of cultural shock—sometimes frivolously, sometimes seriously—on both sides.^48^ Germans believe that the only way to integrate people from different cultures into Germany is to reach out and expose them as much as possible to real life in Germany.

## A FINAL NOTE

Today, we are witnessing the intense civil war in Syria and Iraq, with thousands of innocent people injured or killed and millions fleeing to neighboring countries and to the European Union. Is settlement beyond reach? What does the future hold for the millions who have had to leave everything behind and try to find a new place to start a new life. These are big questions, and no one has all the answers, but we have endeavored in this article to address pertinent issues related to health care generally and cancer management in particular. Our concern with these issues focuses on international recognition of cultural and ethical roles in promoting acceptable solutions for the provision of timely health care to displaced people.

Over the past decade, we have devoted a significant effort to entrench the concept of palliative care in the Middle East.^49-51^ Remarkably, we did not experience a conflict of cultures. As a voluntary regional organization, the Middle East Cancer Consortium has succeeded in building bridges among individuals, communities, and countries in a region undergoing continuous political and military hostilities. Equally important was our endeavor to train professionals over two decades, which has resulted in inspiring a great many medical and governmental organizations throughout the Middle East to acquire the latest updates in cancer care, while bringing together Western and Eastern cultures.
